# Supplementary material for: Quantitative assessment of protein activity in orphan tissues and single cells using the metaVIPER algorithm
Source: Nat Commun. 2018 Apr 16;9:1471. doi: 10.1038/s41467-018-03843-3 (PMC5902599; doi:10.1038/s41467-018-03843-3)
Supplement: Supplementary file 1 — Supplementary Information [file 41467_2018_3843_MOESM1_ESM.pdf]

Quantitative Assessment of Protein Activity in Orphan Tissues and Single Cells Using the metaVIPER Algorithm

Supplementary Information

1 Supplementary Table

Supplementary Table Interactomes used in this work and datasets used to reverse engineer them.

Tissue Type	Expression source	Acronym	# Samples	# Regulators	# Targets	# Interactions	availability
**Bladder urothelial carcinoma**	TCGA RNA-Seq	BLCA	427	6054	19785	489101	aracne.networks
**Breast invasive carcinoma**	TCGA RNA-Seq	BRCA	1212	6054	19359	331919	aracne.networks
**Cervical squamous cell carcinoma and endocervical adenocarcinoma**	TCGA RNA-Seq	CESC	309	6056	19839	583961	aracne.networks
**Colon adenocarcinoma**	TCGA RNA-Seq	COAD	500	6056	19820	413789	aracne.networks
**Esophageal carcinoma**	TCGA RNA-Seq	ESCA	198	5961	18679	529286	aracne.networks
**Glioblastoma multiforme**	TCGA RNA-Seq	GBM	166	6056	19858	563850	aracne.networks
**Head and neck squamous cell carcinoma**	TCGA RNA-Seq	HNSC	566	6055	19772	423104	aracne.networks
**Kidney renal clear cell carcinoma**	TCGA RNA-Seq	KIRC	606	6054	19843	350478	aracne.networks
**Kidney renal papillary cell carcinoma**	TCGA RNA-Seq	KIRP	323	6055	19858	452653	aracne.networks
**Acute myeloid leukemia**	TCGA RNA-Seq	LAML	179	6007	19269	531535	aracne.networks
**Liver hepatocellular carcinoma**	TCGA RNA-Seq	LIHC	423	6056	19829	469922	aracne.networks
**Lung adenocarcinoma**	TCGA RNA-Seq	LUAD	576	6055	19742	399513	aracne.networks
**Lung squamous cell carcinoma**	TCGA RNA-Seq	LUSC	552	6054	19741	455032	aracne.networks
**Ovarian serous cystadenocarcinoma**	TCGA RNA-Seq	OV	299	6007	19140	647358	aracne.networks
**Pheochromocytoma and paraganglioma**	TCGA RNA-Seq	PCPG	187	6506	19861	603617	aracne.networks
**Prostate adenocarcinoma**	TCGA RNA-Seq	PRAD	550	6053	19820	330922	aracne.networks
**Rectum adenocarcinoma**	TCGA RNA-Seq	READ	177	6056	19856	557911	aracne.networks
**Sarcoma**	TCGA RNA-Seq	SARC	265	6112	20479	526591	aracne.networks
**Skin cutaneous melanoma**	TCGA RNA-Seq	SKCM	472	6053	19840	425361	aracne.networks
**Stomach adenocarcinoma**	TCGA RNA-Seq	STAD	307	6056	21663	561858	aracne.networks
**Testicular germ cell tumors**	TCGA RNA-Seq	TGCT	156	6056	19860	432621	aracne.networks
**Thyroid carcinoma**	TCGA RNA-Seq	THCA	568	6053	19861	317582	aracne.networks
**Thymoma**	TCGA RNA-Seq	THYM	122	6056	19862	387923	aracne.networks
**Uterine corpus endometrial carcinoma**	TCGA RNA-Seq	UCEC	581	6055	19716	469845	aracne.networks
**T lymphocyte**	Ref. 1	T	233	5086	13834	324963	figshare
**B lymphocyte**	Ref. 2	B	201	3651	8699	207336	figshare
**Skin cutaneous melanoma**	TCGA RNA-Seq	SKCM	472	6201	19840	432922	figshare
**Glioblastoma multiforme**	REMBRANDT	NA	804	3921	12683	482879	figshare
**Glioblastoma multiforme**	Ref. 3	NA	176	3099	8964	382144	figshare
**Glioblastoma multiforme**	TCGA affymetrix	NA	202	3433	9812	421108	figshare
**Glioblastoma multiforme**	TCGA agilent	NA	202	5560	17355	988514	figshare

2 Supplementary Figures

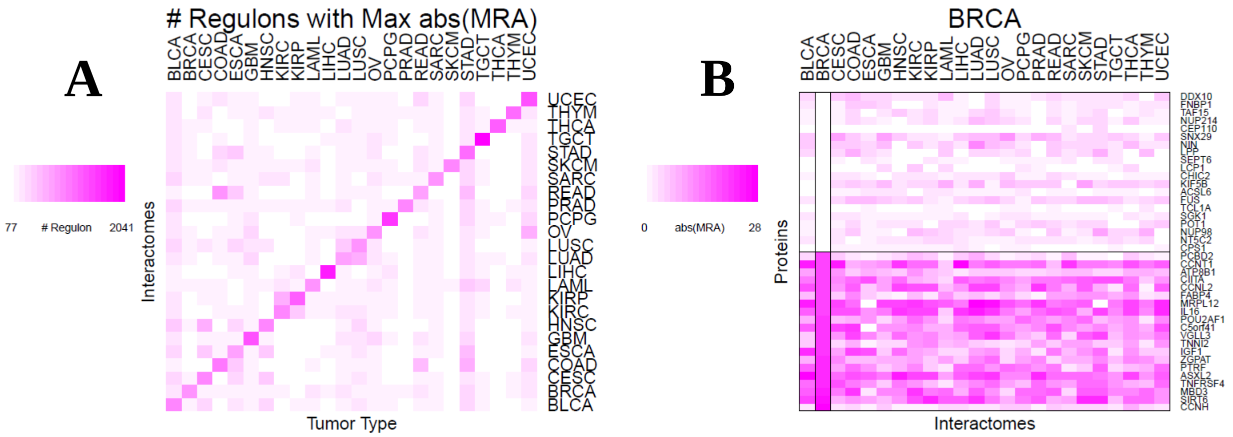

Supplementary Figure 1: Highest absolute NES values are obtained from tissue-matched interactomes. A) Since tissue-matching regulons are in general best models for proteins in that specific tissue, we conclude that correctly assessed regulons usually give high absolute activity. This is demonstrated by that across all tissue types, tissue-matching interactome harbors the most regulons with highest absolute activity. B) In some particular cases, tissue-matching regulons may not constitute the best model. For instance, as shown, in breast invasive carcinoma (BRCA), proteins in the upper panel are best modeled by regulons from other tissues. Also, in some cases, tissue-matching regulons may not be the only appropriate model. For instance, besides BRCA regulons, proteins in the lower panel can also be appropriately modeled by regulons from other tissue types.

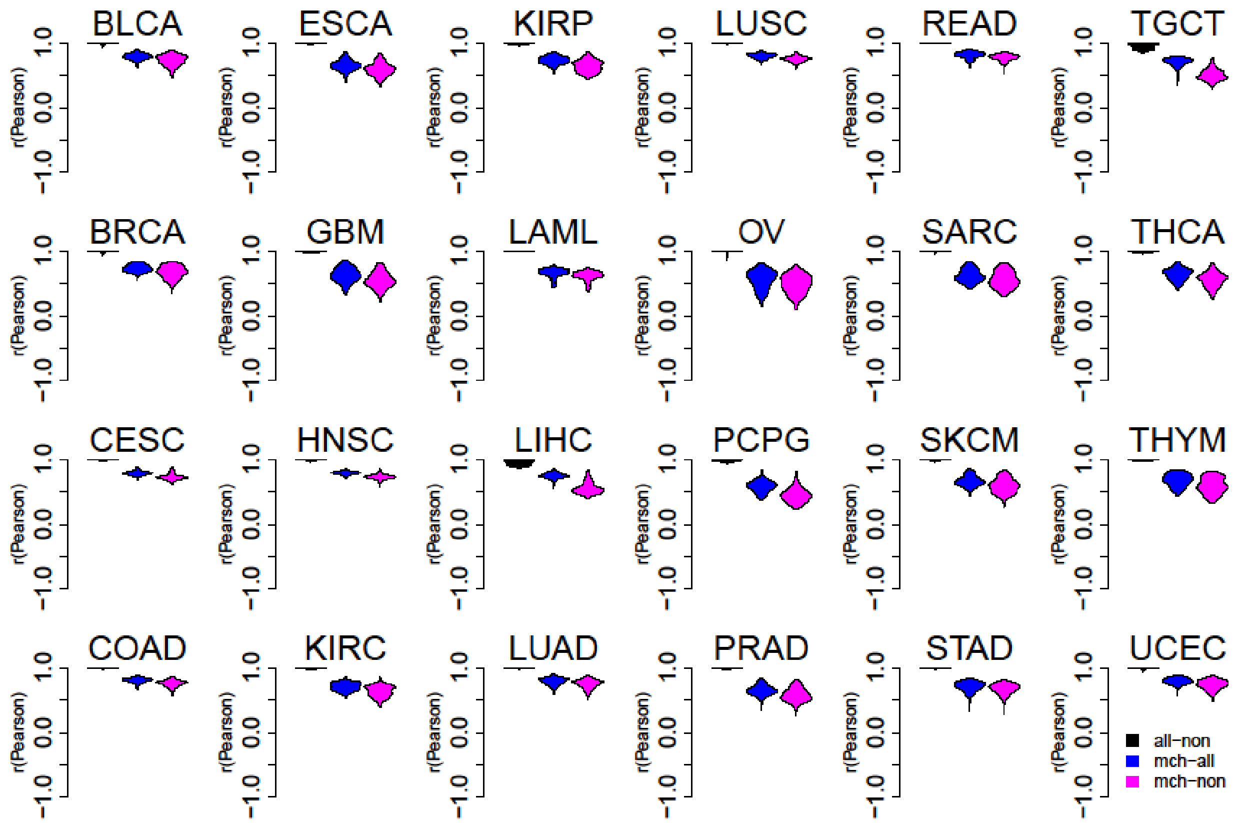

Supplementary Figure 2: Inference protein activity for orphan tissues. Correlation of protein activity inferred from 1) metaVIPER, with all available interactomes (all), 2) metaVIPER, with all non-matching interactomes (non) and 3) VIPER with matching interactome (mch). Then violin plots show the probability density distribution for the Pearson's correlation coefficient for each of the evaluated tissue types.

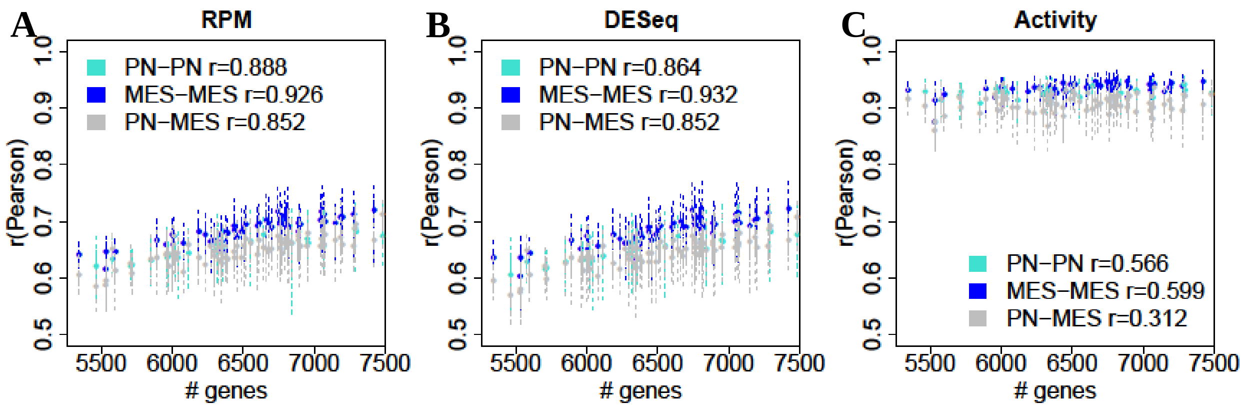

Supplementary Figure 3: Single cell quality as confounding factor in understanding heterogeneity and regulatory properties. With expression measured by both log2(rpm+1) (A) and variance stabilizing transformation (VST) in DESeq R package[4](B), cells with higher quality (higher number of genes detected) tend to have higher correlation with other cells. In some cases, inter-population correlation between high quality cells even exceeds intra-population correlation between low quality cells. This is no longer seen with metaVIPER predicted protein activity (C), indicating it to be a more robust measurement of heterogeneity and regulatory properties from single cells.

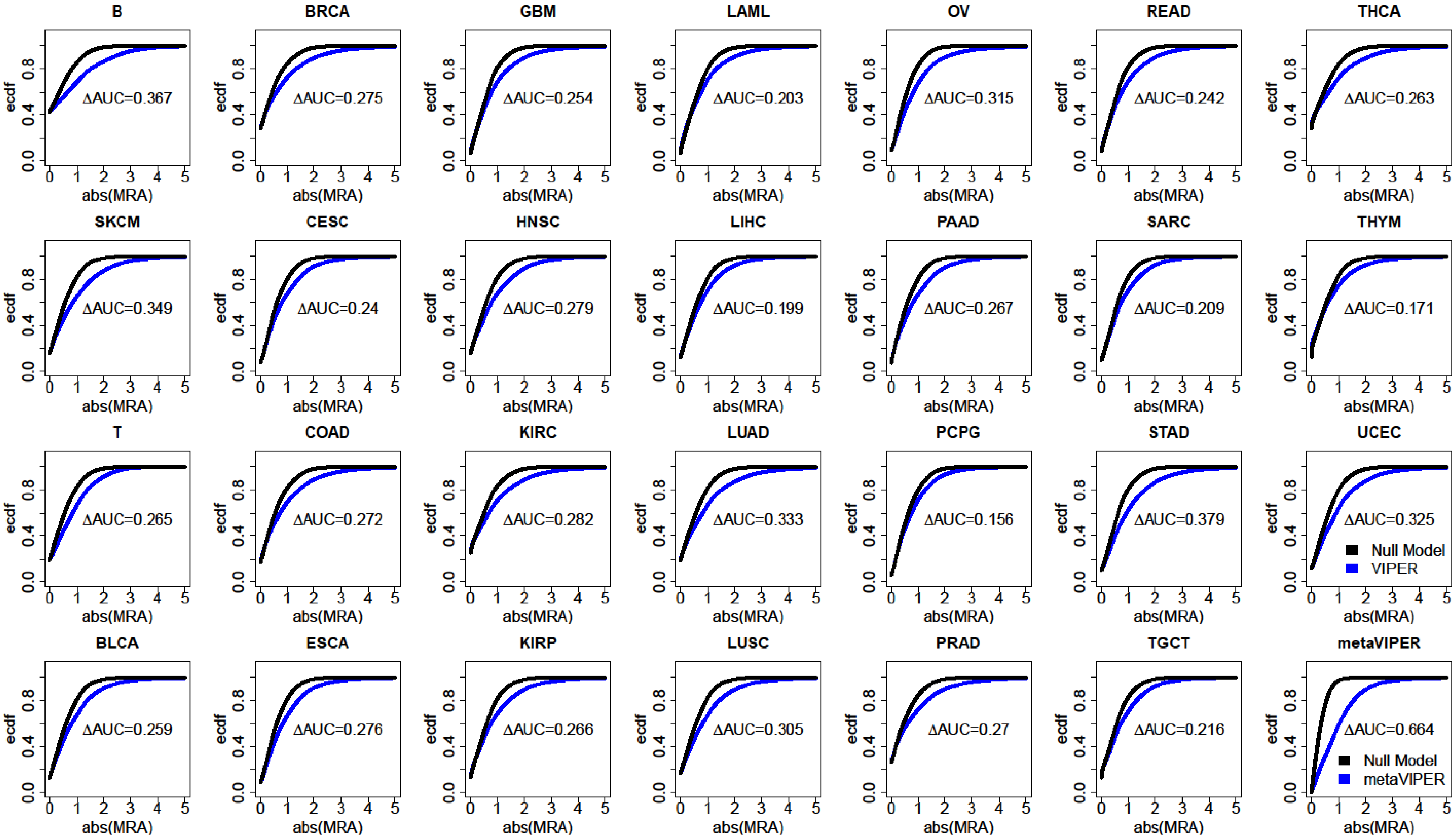

Supplementary Figure 4: metaVIPER integration outperforms analysis with single interactome. As shown in Supplementary Fig 1, for a single cell type, the tissue-matched regulatory model usually gives the highest absolute protein activity inferences. That is to say, in a scenario where different cell types exist, the best regulatory model will give high variance of protein activity across the dataset. We compared the variance of protein activity inferences based on VIPER analysis across 27 distinct tissue-lineage contexts with that of metaVIPER integration. In all cases, metaVIPER outperformed the VIPER analysis. The figures show the ΔAUC between ecdf curve for a null model, built by uniformly shuffling the expression profile sample-wise (shown in black), and that of VIPER/metaVIPER analysis (shown in red), among which metaVIPER gives the highest value.

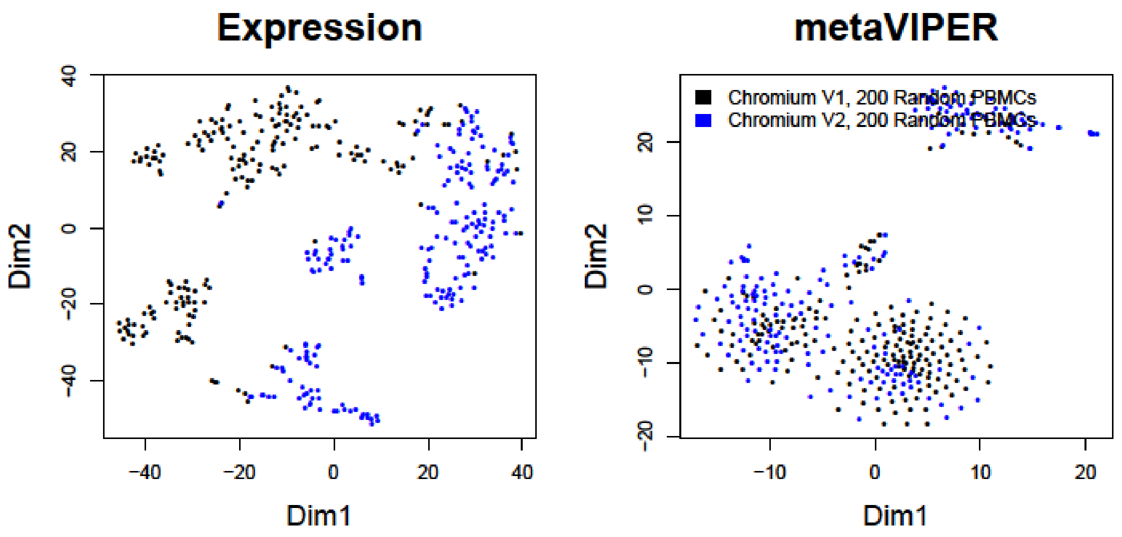

Supplementary Figure 5: metaVIPER reduces discrepancies between different scRNA-Seq data sources. We analyzed filtered PBMC scRNA-Seq data generated using 10x Genomics V1 (Black) and V2 (Blue) chemistry. To make them comparable, we randomly selected 200 single cells for each data sources Discrepancies between different scRNA-Seq data sources were observed using expression, while no longer seen using metaVIPER predicted protein activity

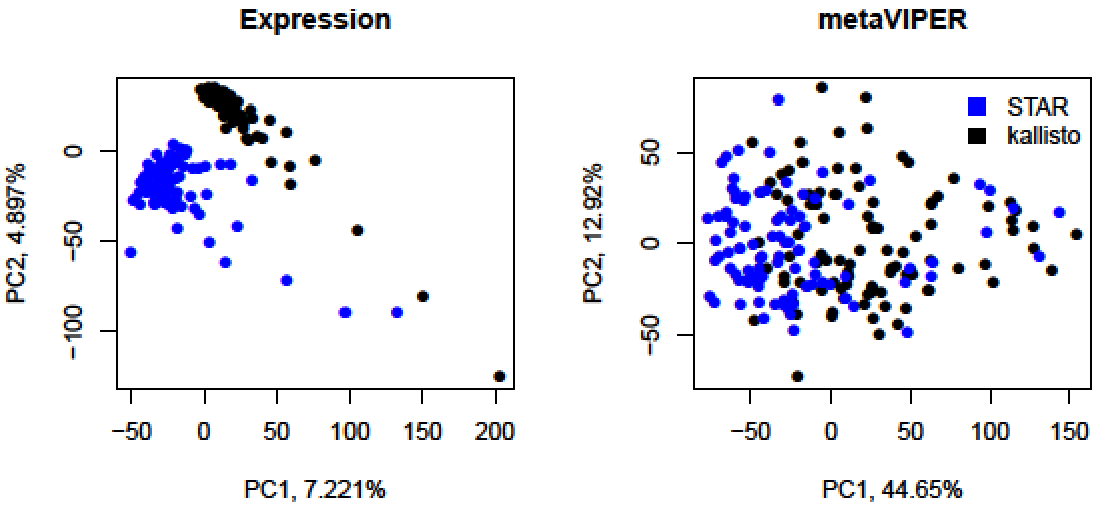

Supplementary Figure 6: metaVIPER reduces discrepancies between different expression quantification tools. We analyzed scRNA-Seq data reported by Wu et al.[5]. Transcriptomic profiles were quantified using STAR[6] (Blue) and kallisto[7] (Black). Discrepancies between different expression quantification tools were no longer seen using metaVIPER predicted protein activity.

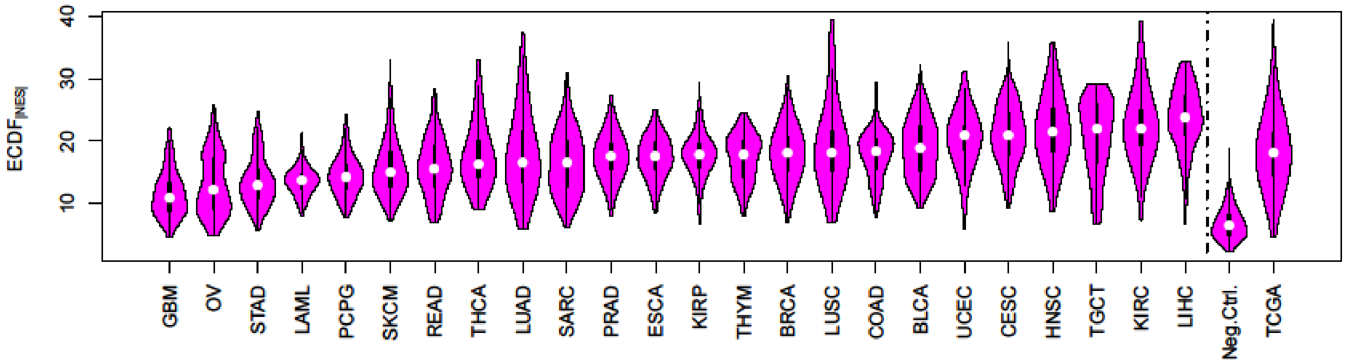

Supplementary Figure 7: Quality control for protein activity analysis. Since properly assigned regulons give high absolute normalized enrichment score (Supplementary Figure 1), therefore we use the *Empirical Cumulative Distribution Function* of the absolute value of the VIPER *Normalized Enrichment Score* ($ECDF_{|NES|}$) of all proteins with significant predicted activity to estimate whether the protein activity analysis is satisfactory. We provided the distribution of the proposed score within each tumor type (GBM, OV etc.) as well as among all TCGA samples (TCGA) using tissue-matching interactome as references for trustworthy protein activity analysis. We also analyzed LAML samples with GBM interactome, which is completely misassigned (LAML and GBM have distinct lineage origination) as the negative control (Neg.Ctrl.). If metaVIPER analysis gives similar result, probably the included interactomes don’t have a satisfactory coverage on regulatory information of the analyzed samples.

References

1 Della Gatta, G. et al. Reverse engineering of TLX oncogenic transcriptional networks identifies RUNX1 as tumor suppressor in T-ALL. Nat Med 18, 436-440 (2012).

2. Lefebvre, C. et al. A human B-cell interactome identifies MYB and FOXM1 as master regulators of proliferation in germinal centers. Mol Syst Biol 6, 377 (2010).

3 Phillips, H. S. et al. Molecular subclasses of high-grade glioma predict prognosis, delineate a pattern of disease progression, and resemble stages in neurogenesis. Cancer Cell 9, 157-173 (2006).

4. Anders, S. & Huber, W. Differential expression analysis for sequence count data. Genome Biol 11, R106 (2010).

5. Wu, A. R. et al. Quantitative assessment of single-cell RNA-sequencing methods. Nature methods 11.1, 41-46 (2014).

6. Dobin, A, et al. STAR: ultrafast universal RNA-seq aligner. Bioinformatics 29.1, 15-21 (2013).

7. Bray, N. L. et al. Near-optimal probabilistic RNA-seq quantification, Nature Biotechnology 34, 525–527 (2016).
